# Alcohol Dependence: Provisional Description of a Clinical Syndrome

**Published:** 1995

**Authors:** Marc Schuckit

**Affiliations:** Marc A. Schuckit, M.D., is professor of pyschiatry, University of California at San Diego School of Medicine, and director of the Alcohol Research Center, San Diego Veterans Affairs Medical Center, San Diego, California

**Keywords:** AOD dependence, diagnostic criteria, disorder definition, AOD withdrawal syndrome

**Figure:**
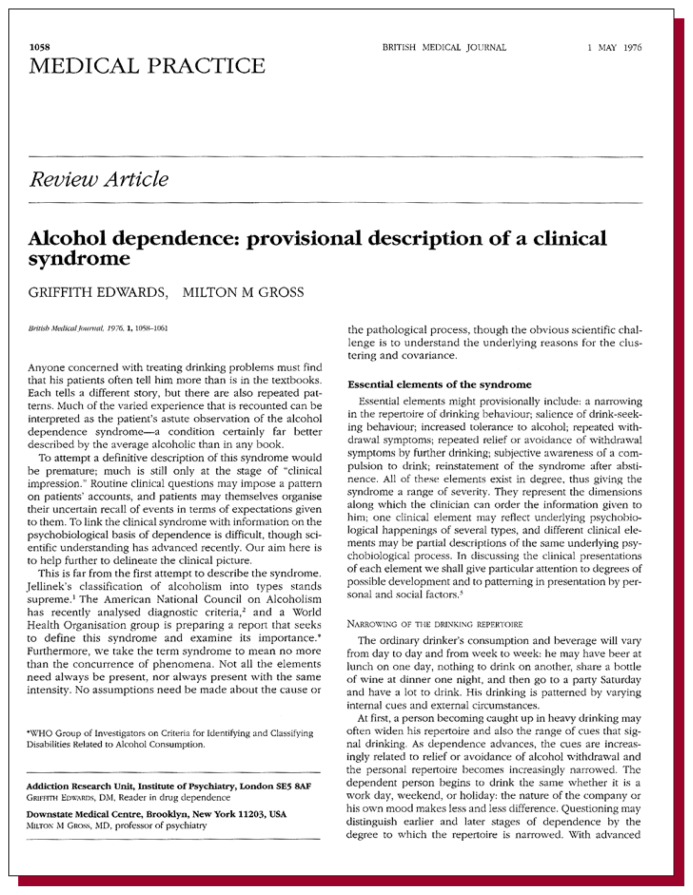
Edwards, G., and Gross, M.M. Alcohol dependence: Provisional description of a clinical syndrome. *British Medical Journal* 6017(1):1058–1061, 1976.

When Edwards and Gross pulished this article in 1976, establishing diagnostic criteria for alcoholism was becoming increasingly important. Much time had passed since Jellinek, in his book *The Disease Concept of Alcoholism* (1960), energized an interest in an area of nosology (the classification of diseases) that had been ignored for centuries. Because of Jellinek’s work, it was no longer necessary to consider alcoholism (also known as intemperance, dipsomania, Folie Alcoolique) as the simple consequence of a lack of willpower or moral fiber and as a condition that was diagnosed solely by the documentation of severe withdrawal symptoms. Attention was now called to a complex interaction between a variety of areas of life impairment, and there was a concomitant recognition by clinicians and researchers that there were likely to be important subgroups of alcoholics with different prognoses and potential treatments.

This renaissance inspired by Jellinek prompted input into diagnostic criteria from behaviorists, scientists interested in learning theory, biologists, and recovering alcoholics. For example, the Washington University criteria, the National Council on Alcoholism criteria, and the Research Diagnostic Criteria were all attempts to define alcoholism more fully. It is in this context that Edwards and Gross published their seminal article, which offered a provisional description of the clinical syndrome of alcohol dependence.

Edwards and Gross’ observations, which pulled together behavioral, learning, and biological theories, were strongly influenced by their involvement in a 1975 meeting of a diagnostic criteria steering group for the World Health Organization. In their article, Edwards and Gross listed seven domains of alcohol-related life experiences that were believed to compose a syndrome or at least what they referred to as a “concurrence of phenomena.” They proposed that most alcohol-dependent men and women will demonstrate some combination of these symptoms, implying that the greater the number of problems, the more intense the severity of the alcoholism. Five of the seven domains were relatively straightforward and fairly easy to implement in clinical practice and research paradigms. These were an increasing salience of alcohol to the lifestyle; evidence of tolerance; the repeated demonstration of withdrawal symptoms (described by Edwards and Gross in impressive detail); the use of alcohol to relieve or avoid withdrawal symptoms; and evidence of a subjective awareness of a compulsion to drink, a concept they described as being akin to a loss of control of alcohol use or perhaps a decision to not exercise control. The two other domains were a bit more difficult to define in objective terms. These were a narrowing of the drinking repertoire (indicating an increasing level of rigidness in the pattern of alcohol use) and a rapid reinstatement of dependence following periods of abstinence.

Even more impressive than Edwards and Gross’ diagnostic algorithm itself (which the authors labeled as provisional and open to modification) is the clinical wisdom set forth in their description of this concept. For example, Edwards and Gross warned that the emphasis should be on the *increasing* priority drinkers give to maintaining their alcohol consumption (not only its overall intensity) to avoid confusing alcohol problems that are related to high levels of impulsivity with problems that are most relevant to a diagnosis of alcohol dependence. The authors also observed that the subjective aspects of a compulsion to drink are highly variable and intermittent, implying that alcohol-dependent men and women are likely to experience limited periods of control over their alcohol intake. Although many alcoholics find abstinence “surprisingly easy to maintain” in specific situations such as on a treatment ward when normal drinking cues are removed, they will begin to drink again later, relapsing into their previous stage of dependence. Rapid reinstatement of alcohol problems after a period of abstinence is accompanied by the caveat that those with very high levels of dependence are likely to never regain control of their drinking. Edwards and Gross were careful to avoid suggesting that there is a rigid progression of alcohol problems leading to dependence that applies to everyone. Instead, they suggested that the degrees of the dependence syndrome, as with most syndromes, are “shaped and colored by personality and environment.”

Elements of the dependence syndrome as defined by Edwards and Gross found their way into the official diagnostic systems with the 1977 publication of the ninth version of the *International Classification of Diseases* (ICD–9). The 1980 American Psychiatric Association’s *Diagnostic and Statistical Manual of Mental Disorders, Third Edition* (DSM–III), began to move toward Edwards and Gross’ concept by recognizing at least two types of alcoholism, abuse and dependence, and demonstrating that people with alcohol problems did not necessarily have to show a tolerance to alcohol or experience withdrawal symptoms to receive a diagnosis of alcoholism.

The natural evolution of these diagnostic systems culminated in the third revised version of the *Diagnostic and Statistical Manual* in 1987 (DSM–III–R), whose description of alcoholism was based primarily on Edwards and Gross’ definitions of dependence. The most recent versions of both manuals, published as ICD–10 (1992) and DSM–IV (1994), are now in close agreement on this definition of dependence (although they diverge rather widely on the criteria for the less intense alcohol-related phenomenon, abuse or harmful use).

Although animal models and some clinical data do exist that offer general support for the existence of the dependence syndrome as defined by Edwards and Gross, there is no definitive research that supports such a complex concept. However, both DSM–IV and ICD–10 pay homage to the clinical usefulness of the Edwards and Gross approach. For example, as part of the process of preparing DSM–IV for publication, a field trial was performed using more than 1,000 subjects. It compared the clinical coverage (i.e., the proportion of impaired subjects who received a diagnosis) and the clinical correlates of the dependence syndrome with alternative versions of a diagnosis of alcoholism; the Edwards and Gross approach for defining dependence was at least as good as any other diagnostic system for defining alcoholism that was evaluated. This description of alcoholism proposed by Edwards and Gross and used in DSM–IV has additional benefits. The dependence syndrome could be applied to drugs other than alcohol, components of the dependence approach could be tested in both animals and humans, and there are some indications that severity of dependence might be related to the number of problems identified.

In considering Edwards and Gross’ article from the perspective of the two decades that have passed since its publication, it is clear that it continues to occupy a pivotal position in the alcohol field. The authors have not produced an inviolable dictum but offer an exceptionally useful provisional description of a clinical syndrome. Edwards and Gross point out that additional research is required on the optimal definition of each of the concepts they outline as well as on the appropriate cutoff point for demonstrating the existence of pathology. Research also is necessary to explore alternative definitions of subgroups based on age, gender, and cultural background, and work is underway to attempt to understand more about the social, biological, and learning model components of the dependence syndrome.

In summary, this article deserves this accolade, which others in the alcohol field can only hope might someday be applied to their work: The field has been significantly enriched by its publication.
